# Next-generation sequencing reveals novel variants and large deletion in *FANCA* gene in Polish family with Fanconi anemia

**DOI:** 10.1186/s13023-022-02424-4

**Published:** 2022-07-19

**Authors:** Anna Repczynska, Katarzyna Julga, Jolanta Skalska-Sadowska, Magdalena M. Kacprzak, Alicja Bartoszewska-Kubiak, Ewelina Lazarczyk, Damian Loska, Malgorzata Drozniewska, Kamila Czerska, Jacek Wachowiak, Olga Haus

**Affiliations:** 1 Department of Clinical Genetics, Faculty of Medicine, Collegium Medicum in Bydgoszcz, Nicolaus Copernicus University, Toruń, Poland; 2 Department of Pediatric Oncology, Hematology and Transplantology, University of Medical Sciences, Poznan, Poland; 3 Medgen SA, Warsaw, Poland; 4 West Midlands Regional Genetics Laboratory, Birmingham Women’s and Children’s Hospital NHS Foundation Trust, Birmingham, UK

**Keywords:** Fanconi anemia, Thrombocytopenia, Chromosome breakage test, aCGH, *FANCA* gene

## Abstract

**Background:**

Fanconi anemia (FA) is the most common inherited bone marrow failure syndrome. However, establishing its molecular diagnosis remains challenging. Chromosomal breakage analysis is the gold standard diagnostic test for this disease. Nevertheless, molecular analysis is always required for the identification of pathogenic alterations in the FA genes.

**Results:**

We report here on a family with FA diagnosis in two siblings. Mitomycin C (MMC) test revealed high level of chromosome breaks and radial figures. In both children, array—Comparative Genomic Hybridization (aCGH) showed maternally inherited 16q24.3 deletion, including *FANCA* gene, and next generation sequencing (NGS) disclosed paternally inherited novel variants in the *FANCA* gene—Asn1113Tyr and Ser890Asn. A third sibling was shown to be a carrier of *FANCA* deletion only.

**Conclusions:**

Although genetic testing in FA patients often requires a multi-method approach including chromosome breakage test, aCGH, and NGS, every effort should be made to make it available for whole FA families. This is not only to confirm the clinical diagnosis of FA in affected individuals, but also to enable identification of carriers of FA gene(s) alterations, as it has implications for diagnostic and genetic counselling process.

## Background

Fanconi anemia (FA) is the most common form of inherited bone marrow failure syndromes (IBMFS) related to developmental abnormalities. Clinical symptoms, present in about 75% of patients, most often include short stature, microcephaly, thumb and radial side limb defects, abnormal skin pigmentation, and genitourinary defects. Progressive bone marrow failure occurs in the first decade of life, initially often expressed by leukopenia or thrombocytopenia. The most common malignancies occurring in patients with FA are acute myeloid leukemia and solid tumors of the head and neck, skin, and gastrointestinal as well as genitourinary systems [1, 2]. At the cellular level, FA is characterized by sensitivity to interstrand crosslinking agents (ICL), such as diepoxybutane (DEB) and mitomycin C (MMC) [3, 4]. FA is mostly caused by biallelic pathogenic variants in any of the 21 genes: *FANCA, FANCC, FANCD1(BRCA2), FANCD2, FANCE, FANCF, FANCG, FANCI, FANCJ(BRIP1), FANCL, FANCM, FANCN(PALB2), FANCO(RAD51C), FANCP(SLX4), FANCQ(ERCC4), FANCS(BRCA1), FANCT(UBE2T), FANCU(XRXX2), FANCV(MAD2L2/REV7), FANCW(RFWD3), FANCY(FAP100).* Variants in *FANCB (*presenting X-linked recessive inheritance pattern) and *FANCR/RAD51 (*presenting autosomal dominant inheritance pattern) have also been reported as disease-causing, therefore a total of 23 genes have been implicated in the pathogenicity of FA so far [5, 6]. In two-thirds of FA patients pathogenic variants occur in *FANCA*, making it the most frequently mutated FA gene. Approximately 20% of patients carry mutations in *FANCC* or *FANCG,* and mutations in the other 20 FA genes account for 0.1–4% cases per gene. *FANCA* variants include single nucleotide variants, small insertions, and deletions which may be detected by Next Generation Sequencing (NGS). However, 20–40% of *FANCA* mutations are large deletions, which size span a wide range from ~ 1 to 545 kb, identified by array—Comparative Genome Hybridization (aCGH) or multiplex ligation-dependent probe amplification (MLPA) [7, 8].

The wider study we performed under the research grant WL131 aimed to provide a detailed and accurate genetic diagnosis for all Fanconi anemia individuals and their families by establishing a comprehensive diagnostic approach for this rare condition. In this paper, we focus on clinical presentation and the results of genetic analyses in one selected family showing that a broad approach comprising chromosome breakage test followed by chromosomal microarray analysis and NGS is not only sufficient to provide the genetic diagnosis in FA patients, but also proves beneficial when extended to the whole family.

## Methods

### Clinical and hematological report

**Fig. 1:**
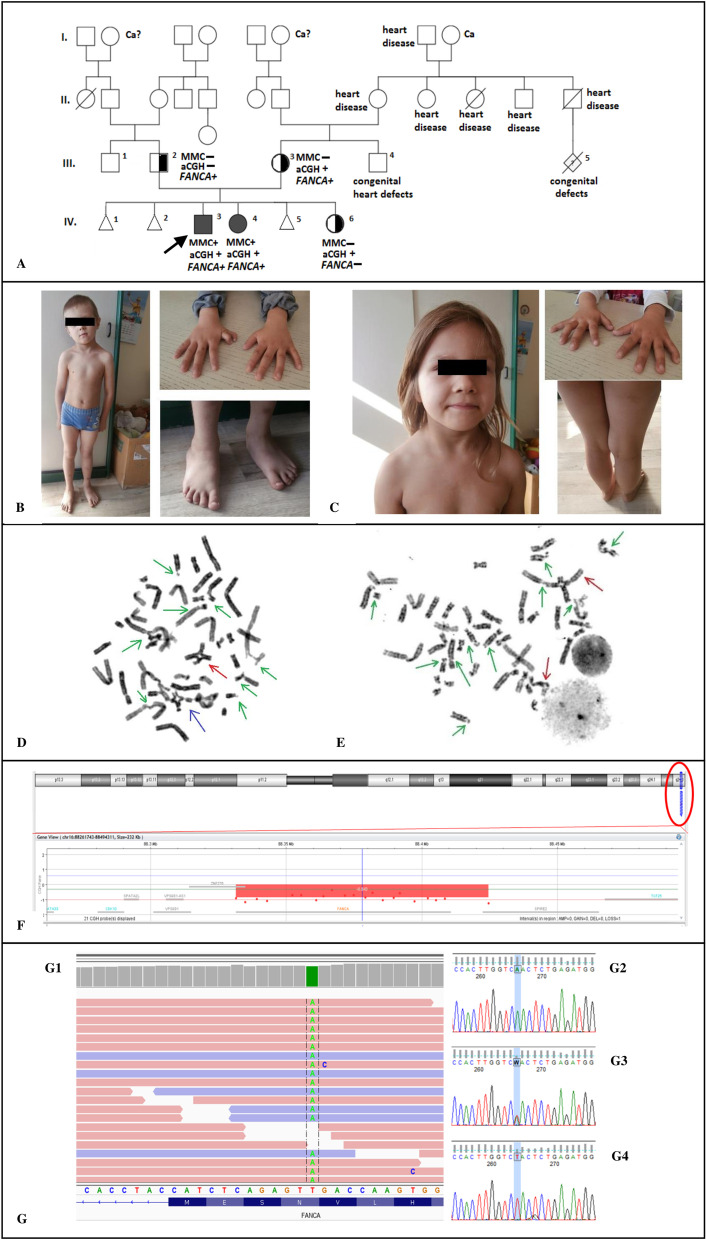
Summary of clinical and genetic findings in the presented family. **a** Pedigree of FA family. Squares—males, circles—females, open—unaffected individuals, filled—affected individuals, half filled—carriers of deletion or mutation. MMC+/− = positive or negative result of MMC test, aCGH+/− = presence or absence of heterozygous deletion encompassing *FANCA* gene detected by aCGH, FANCA+/− = presence or absence of mutation in *FANCA* gene detected by NGS, Ca/Ca? = presence or possible presence of cancer. Black arrow indicates the proband. **b**–**c** Congenital anomalies in siblings with FA. B—short stature, posture defect, extra thumb and pes plano-valgi in patient 1 (IV.3). C—large short neck, corrected extra thumb, and cafe-au-lait spots in patient 2 (IV.4). **d**–**e** Metaphase spreads in patients 1 (**d**) and 2 (**e**)—MMC test. Green arrows show gaps, chromatid breaks (chtb) and acentric fragments (ace). Red arrows show radial figures. Blue arrow shows chromatid interchange figure. **f** Partial array-CGH result showing the 16q24.3 deletion in the proband (patient IV.3). The same heterozygous deletion was detected in his siblings and mother. **g** Visualisation of *FANCA* sequencing reads showing c.3337A > T; p.Asn1113Tyr variant (g1), and Sanger sequencing electrophoregram plots confirming the variant (g2—wild type found in the mother III.3, and younger daughter IV.6, g3—mutation in the heterozygous father III.2, g4—mutation in the only copy of the gene in IV.3 and IV.4 siblings)

A 5-year-old male (Patient 1—IV.3) was born with a birth weight of 2520 g, and Apgar score of 10. He was the first child of unrelated healthy parents. There was a history of three recurrent miscarriages in his mother, as well as a presence of congenital defects and early infant death on the maternal side of the pedigree (Fig. 1a). An extra thumb, a heart defect with patent ductus arteriosus (PDA), and abnormal localization of the right kidney were noted at newborn period. PDA was closed with coil implementation at the age of 6 months. During the following years short stature, low weight, café-au-lait spots, and conductive hearing impairment became apparent. At the age of five he was referred to the Clinical Genetic Department where slight epicanthic folds, large and short neck, kyphoscoliosis, pes plano-valgi were also noted (Fig. 1b). The boy met the criteria of congenital abnormality score (CABS) 3 (1 point each for heart anomaly, kidney defect, and hearing impairment) [9].

For the first 2 years of life complete blood count was normal except for the episode of acute hemolytic anemia due to the PDA (this presented with elevated unconjugated bilirubin levels, reticulocyte count, and decreased haptoglobin). Complete recovery was achieved after additional coil deployment and infusion of 7 units of red blood cell concentrate.

The next episode of cytopenia occurred at the age of 2 years with decreased platelet count on pneumonia. In the third year of life the median platelet and neutrocyte counts were 110 × 10^3^/µl (91–129) and 1 × 10^3^/µl (0.98–2.8), respectively. In the fourth year of life these results amounted to 78 × 10^3^/µl (35–103) and 1 × 10^3^/µl (0.67–1.5), in the fifth one to 58 × 10^3^/µl (35–99) and 0.95 × 10^3^/µl (0.53–1.9), respectively. For the first time hemoglobin reduction to 9.8 g/dl occurred at 6 years of age, and at the same time platelet count dropped below 20 × 10^3^/µl. The boy started to demonstrate hemorrhagic symptoms (bruising, epistaxis) and became dependent on platelet transfusions. Myelogram showed low cellularity (20–30%) represented nearly exclusively by the erythroid lineage cells.

**Fig. 2:**
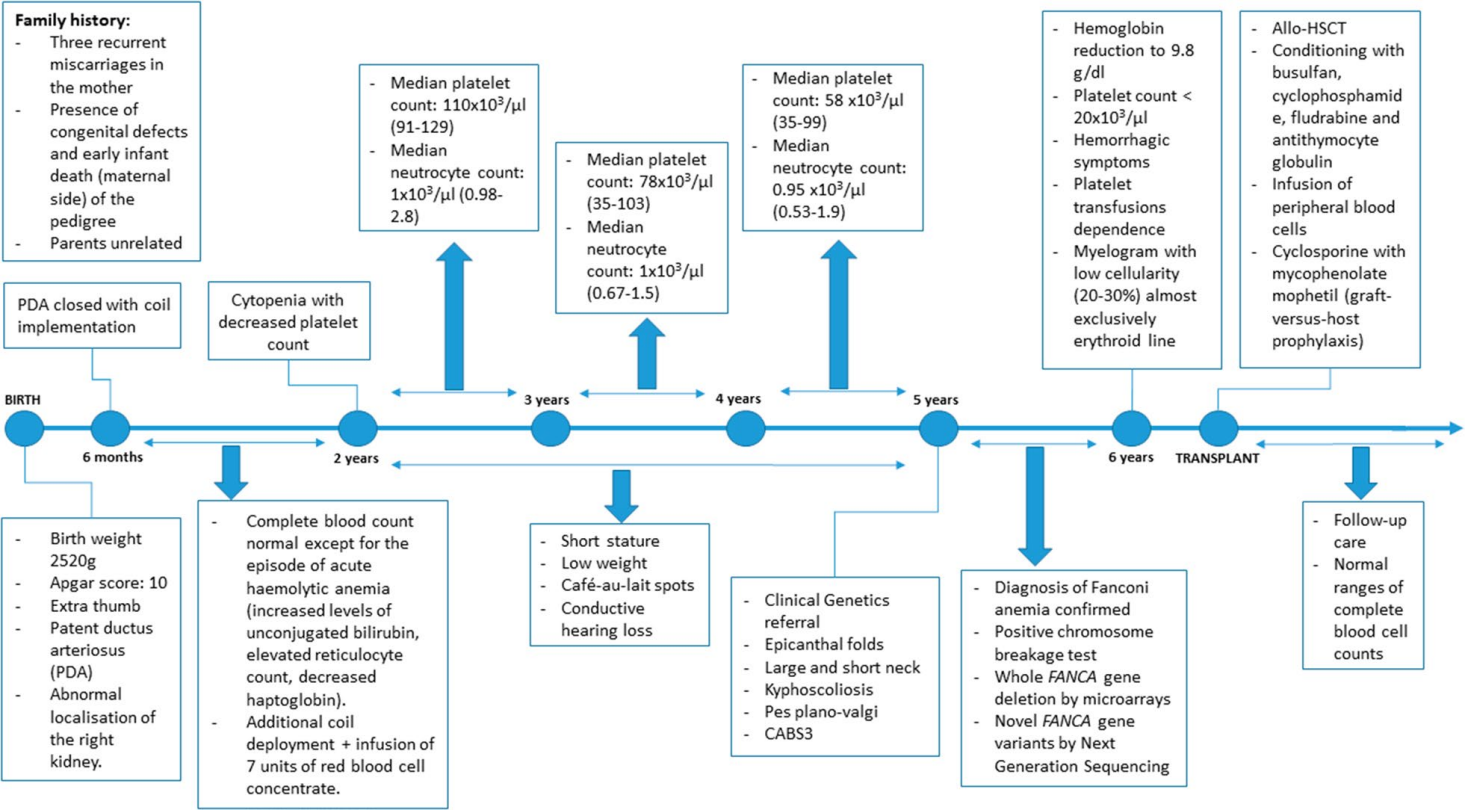
Timeline summary of clinical and diagnostic data for patient IV.3 (proband)

After the diagnosis of FA was confirmed by genetic testing, an allogeneic hematopoietic stem cell transplantation (allo-HSCT) was performed from the matched unrelated donor (MUD). Following conditioning with busulfan (0.8 mg/kg/day, 4 days), cyclophosphamide (10 mg/kg/day, 4 days), fludarabine (30 mg/m^2^/day, 5 days) and antithymocyte globulin (40 mg/kg/day, 4 days), peripheral blood cells of 4.5 × 10^6^ CD34 + /kg and 0.87 × 10^8^ CD3 + /kg were infused. Cyclosporine (days − 3 to + 100) with mycophenolate mophetil (45 mg/kg/day days + 1) for graft-versus-host disease (GvHD) prophylaxis was administered. The time to neutrophil (> 0.5 × 10^9^/l), platelet (> 20 × 10^9^/l) engraftment was 14 and 9 days, respectively. The first reticulocytes emerged on day + 10, and maximal reticulocyte count (41‰) was achieved on day + 17, with complete donor chimerism. Only minor feverish episodes complicated the post-transplant course. There were neither symptoms of viral infections reactivation nor graft versus host disease. The boy receives regular follow-up care, and up to date presents with normal ranges of a complete blood cell counts. Timeline summary of clinical and diagnostic information for this patient is shown in Fig. 2.

His 1 year younger sister (Patient 2—IV.4) was born with a birth weight of 2325 g and Apgar scores of 10. An extra thumb, PDA, and ectopic pelvic left kidney were noted at the newborn period. Due to suspicion of Fanconi anemia in the family, the girl was referred for genetic evaluation. On physical examination a large philtrum, large short neck, and large chest were noted (Fig. 1c). The girl fulfilled the criteria of CABS2 score. She shows normal growth parameters according to standard growth curves, her PDA is stable and does not require surgical intervention. The complete blood count has been monitored since the girl was five. In her sixth and seventh years of life the median neutrocyte count was 1.4 × 10^3^/µl, while the median platelet count was 128 × 10^3^/µl and 97 × 10^3^/µl, respectively.

The youngest sister (Patient 3—IV.6) and the parents have not presented symptoms similar to those of older siblings.

### G-banding analysis and chromosome breakage test

Heparinized venous blood (≥ 5 ml) was used to prepare whole-blood cultures as usual for routine cytogenetic analysis. Four cultures per individual and healthy control were prepared by adding 0.5 ml blood to 4.5 ml complete RPMI medium (Gibco) supplemented with 15% fetal bovine serum (Gibco), gentamicin and phytohemagglutinin (PHA) (both Gibco), as prescribed (Joenje and co-workers). The cultures were incubated for 72 h with 0, 50, 150, or 300 nM mitomycin C (MMC; Sigma Aldrich). After treatment with colcemid (0.1 µg/ml) for 50 min, cells were harvested, incubated with 0.075 M KCL for 20 min at 37 °C, and fixed with 75% methanol and 25% acetic acid. Cytogenetic preparations were stained for 2 min in a 5% Giemsa solution (Merck); banding technique was applied only to slides from cultures without MMC to analyze the karyotype. The microscope analysis was performed with a Nikon E600 microscope and later analyzed by computer assisted metaphase system (ASI, Israel).

Chromosome instability data were analyzed and calculated (number of metaphases with breakage, mean chromosome break events per aberrant metaphase, and tri-, tetra-, and multi- radials frequency) [10]. A healthy control was assured.

### Nucleic acid extraction

Blood samples were collected from the proband, his sisters and parents. Whole genomic DNA was extracted from nucleated cells by silica gel column methods according to the manufacturer ‘s protocol (Qiagen) for aCGH assay and by using Prepito TM for NGS assay.

### Array—Comparative Genomic Hybridization (aCGH)

aCGH was performed using the commercially available platform (Sure Print G3 Human CGH 8 × 60Microarray Kit, Agilent Technologies, Santa Clara, CA, USA), according to the manufacturer's protocol. Copy number analysis was performed using Agilent CytoGenomics Software 5.0.

### Next generation sequencing (NGS)

A targeted next-generation sequencing (MiSeq, Illumina) was performed using a **targeted** multigene **custom** panel including *ATM, BLM, BRIP1, FANCA, FANCC, FANCG, NBN*, and *PALB2* genes. The sequencing library preparation was performed using SeqCap EZ **Choice** NimbleGen (Roche). The sample was sequenced with Illumina technology on Miseq sequencer with 2 × 75 bp reads. Demultiplexing of the sequencing reads was performed with Illumina's bcl2fastq2 v2.19.0. Adapters were trimmed with Skewer version 0.2.9 [11]. The reads were aligned to GRCh37/hg19 reference sequence using BWA-MEM [12]. Read duplicates were removed using Picard 2.18.2 (http://broadinstitute.github.io/picard/). Variant call was performed with GATK v4.0.3.0 HaplotypeCaller [13, 14] and FreeBayes (v1.2.0-2-g29c4002) [15]. Variants have been annotated with databases (i) VEP97 [16]: annotations Sift, Polyphen2, (ii) dbNSFPv4.0 [17] annotations: MutationAssessor, MutationTaster, DANN, FATHMM, (iii) ESP6500, (iv) GnomAD, (v) dbSNP, (vi) ClinVar and (vii) 1000 Genomes [18].

For the purpose of the study all the genetic analyses were performed in all siblings and their parents.

## Results

### G-banding analysis and chromosome breakage test

Cytogenetic GTG analysis showed normal karyotypes in all family members.

A standard chromosome breakage test with mitomycin C revealed chromosomal hypersensitivity to crosslinking agent in patients 1 (IV.3) and 2 (IV.4) (Fig. 1d–e). The number of metaphases with breaks, the mean number of breaks per aberrant metaphase, and radials frequency were higher than the normal range for non-FA cells (Table 1). The cytogenetic findings were compatible with Fanconi anemia. The results of patient 3 (IV.6) and the parents were normal.

**Table 1:** Cytogenetic findings of spontaneous and MMC-induced chromosome aberrations (breaks, radial forms) of examined patients 1 (IV.3) and 2 (IV.4) compared to healthy control group

	Spontaneous	MMC induced
*Patient 1*		
Total cells	22 cells	50 cells
% Cells with breaks	14.00%	96.00%
Breaks/cell	0.14 br/cell	8.48 br/cell
Radial forms	None	65
*Patient 2*		
Total cells	21 cells	50 cells
% Cells with breaks	0.00%	100.00%
Breaks/cell	0.00 br/cell	10.84 br/cell
Radial forms	None	107
*Control*		
Total cells	20 cells	50 cells
% Cells with breaks	0.00%	4.00%
Breaks/cell	0.00 br/cell	0.05 br/cell
Radial forms	None	None

*br* breaks

### Array—Comparative Genomic Hybridization (aCGH)

In patients 1 (IV.3), 2 (IV.4), and 3 (IV.6), aCGH analysis showed a heterozygous deletion of ~ 93 kb in chromosome 16q24.3 (Fig. 1f), encompassing the whole FANCA gene (arr[hg19] 16q24.3(89804014_89897040) × 1). Parental array analysis showed the same heterozygous deletion in the mother (III.3). No other copy number imbalances of potential clinical significance have been detected.

### Next generation sequencing (NGS)

Next-generation sequencing analysis revealed in patients IV.3 and IV.4 the presence of three rare variants (with minor allele frequency < 1%), all localized in *cis*, in the only allele of *FANCA* gene: (i) a novel variant c.3337A > T; p.(Asn1113Tyr) found in exon 33 [NM_000135.2; NP_000126.2] (Fig. 1g1); (ii) c.2669G > A; p.(Ser890Asn), rs1055448646 in exon 28, and (iii) c.1614G > A; p.(Gly538 =), rs747421581 in exon 17. According to ACMG classification [19], the p.**Asn1113Tyr** is a likely pathogenic variant—it meets the criteria of PM1 (is located in a mutational hotspot and/or critical and well-established functional domain), PM2 (is absent in mutation databases such as gnomAD), PP2 (missense variants are a common cause of the disease), PP3 (bioinformatic algorithms predict its pathogenic character). p.**Ser890Asn**, fulfilling PM2 (low populational frequency), PP2, and BP4 (bioinformatic algorithms predict its benign character) criteria is classified as VUS (Variant of Uncertain Significance). The synonymous variant p.**Gly538 = ,** fulfilling PM2, BP4, BP6 (reputable source recently reports variant as benign but the evidence is not available to the laboratory to perform an independent evaluation), and BP7 (a synonymous variant for which splicing prediction algorithms predict no impact to the splice consensus sequence nor the creation of a new splice site AND the nucleotide is not highly conserved) criteria is likely a benign variant. The presence of all variants was confirmed by Sanger sequencing (Fig. 1g2–4.), revealing their paternal origin. Table 2 shows summary of detected variants.

**Table 2:** Clinically relevant molecular findings in *FANCA* gene in examined family

Patient	Asn1113Tyr	Ser890Asn	Large deletion	Genotype
IV.3/brother	+	+	+	m/del
IV.4/older sister	+	+	+	m/del
IV.6/younger sister	−	−	+	−/del
III.3/mother	−	−	+	−/del
III.2/father	+	+	−	m/−

*m* mutation, *del* deletion

To confirm the pathogenic character of *FANCA* gene variants identified in affected children and their father, functional studies should be performed. Our classification of mutations done in accordance with mutation databases has only presumable character.

## Discussion

Fanconi anemia is a complex disorder, therefore understanding its underlying molecular mechanisms is critical not only for the diagnosis but also for the clinical management of the affected individuals and their families [20].

Clinical presentation of FA patients is highly variable even among individuals within the same family or among patients within the same complementation group, therefore the diagnosis should be confirmed by a positive chromosomal breakage test and identification of a causal pathogenic mutation in one of the FA-related genes [21, 22].

The *FANCA* gene (OMIM: 607139) is located on chromosome 16q24.3, contains 43 exons along a coding sequence of 4.3 kb, spans approximately 80 kb [23, 24] and is by far the most commonly mutated gene in patients with FA worldwide, accounting for the disease in the 60–70% of FA families. However, the percentages may differ for certain ethnic populations [8, 22, 25–29]. Therefore, molecular testing usually starts with this particular gene [8, 29]. Extensive allelic and non-allelic heterogeneity of *FANCA* gene mutations causes major problems in molecular diagnostics. If no mutation in *FANCA* is detected, the screening is extended to other FA genes [31]. The next most frequently mutated genes that are responsible for ~ 10–15% of cases are *FANCC* and *FANCG* [8, 22, 27, 28]. Causative mutations in other FA genes are rare and account for 0.1–3% of cases per gene [8, 22].

Several diagnostic approaches for FA have been described in the literature. The diagnosis of FA is still classically based on clinical evaluation followed by exposing patients’ lymphocytes to MMC or DEB, and performing subsequent quantification of all types of chromosomal breakages and radial forms [29, 30]. Although a positive chromosome breakage test is highly indicative for FA, it is not 100% specific for FA and can produce false-negative and false-positive results [31, 32]. Therefore, a molecular investigation is needed for definitive and accurate diagnosis, prognosis assessment and genetic counseling for FA families [28]. This, however, can be a daunting, complex, and time-consuming task [8, 20, 23, 29] due to genetic heterogeneity, large (and constantly growing) number of FA genes, together with a wide spectrum of private mutations [33].

Complete molecular diagnosis of the disease-causing specific pathogenic variants is fundamental for confirmation of the diagnosis, management of patients, genetic counseling, carrier testing, and prenatal diagnosis. Molecular diagnosis is also critical for the identification of appropriate donors for bone marrow transplantation, and for exploring novel therapeutic approaches [7, 8, 29, 31].

FA-related genes present a remarkable mutational heterogeneity [26]. The ever-growing portfolio of mutations includes point mutations, small insertions/deletions, splicing defects, and large deletions [8, 34]. The latter appears to be a frequent subtype of variation, especially in *FANCA*, accounting for 20–40% of all pathogenic variants of this gene [7, 8, 22, 35, 36].

Significant proportion of large deletions results from a high content of *Alu* repeats in this region. *Alu* repeats can facilitate intrachromosomal recombinations and generate intragenic copy number variants through unequal crossing-over and their presence is directly correlated with deletion breakpoints [28, 29, 36–38]. *Alu* elements can also induce exon skipping with the effect of both upstream and downstream alternative exons [23, 35].

Several methods have been used in screening protocols for *FANCA.* However, it became clear that they must incorporate methods that can detect point mutations as well as small and large deletions [26, 36]. In the past, after clinical diagnosis and a positive chromosome breakage test, FA patients' molecular characterization was achieved by Sanger sequencing of candidate genes. This approach for FA is time-consuming, costly and, the most importantly, may not detect all types of disease-causing mutations [21]. NGS seems to facilitate a more comprehensive diagnostic strategy for FA patients' characterization, especially with the development of targeted panels for FA genes [22, 26].

Large deletions can be detected by multiplex ligation-dependent probe amplification assay [8]. However, MLPA is a targeted assay and can identify only indicated DNA sequences. Moreover, it identifies only deleted exons, but not the precise breakpoints [7]. Until now, there has been very little effort made to expand the screening for all FA genes at once and to define the precise molecular nature of particular deletions. *FANCA* deletion analysis by MLPA was previously proposed as an initial step in a comprehensive mutation screening strategy [8, 21, 35]. Moreover, 90% of all FA gene deletions occur in *FANCA*, and approximately half of them extend beyond the *FANCA* gene region, affecting the integrity of neighboring genes and influencing patient's phenotype [7, 8, 35]. Thus, the larger insight in the phenotypic (including clinical) picture of the patients is needed.

Currently, aCGH can be considered as an integral component of a comprehensive strategy for identifying disease-causing variants in FA genes. Efforts should be made to correlate these variants with associated phenotypic changes. The identification of precise breakpoints possible by aCGH in contrast to MLPA, allows for quick screening of deletions in family members and provides insight into the deletion driving mechanisms [7, 8].

In our study, we present a family with compound alterations in *FANCA* gene.

In all children, a heterozygous deletion of chromosome region 16q24.3 was detected, including the entire *FANCA* gene. The same deletion was present in the mother. As this heterozygous deletion did not explain clinical features in patients IV.3 and IV.4, mutational analysis using next generation sequencing was performed, which identified the presence of a novel paternally inherited c.3337A > T; p.(Asn1113Tyr) pathogenic variant in exon 33 of *FANCA* gene accompanied by c.2669G > A; p.(Ser890Asn) variant, classified as VUS (variant of uncertain significance). To finally determine the meaning of each variant, it is necessary to perform functional tests.

This study has its limitations. Functional studies, together with protein modelling, were outside the initial scope of this study and its funding. These will, however, be performed in a follow-up project, and the results will be made available separately.

In the era of precision medicine, detailed diagnostic work-up and genetic testing are of great importance for accurate diagnosis, prognostic estimation, and personalized treatment, especially for rare and life-threatening disorders.

## Conclusions

Our study adds to observations made by other authors, that the application of evolving new technologies can help mutation detection in genetically heterogeneous diseases become more economical, affordable, and efficient. Mutation identification in a FA patient, and establishing a carrier status in relatives, is an important part of the diagnostic and genetic counselling process for each FA family [7, 20, 25].

Genetic testing is a valuable tool for families with FA suspicion. There are several reasons for it. First, it confirms the diagnosis of FA among patients with inconclusive chromosome breakage tests or those with clinical features overlapping with other genetic syndrome features. Second, the identification of the family mutations could be useful for prenatal screening in future pregnancies. Third, heterozygous carriers could be identified within the family with FA and the genetic counseling could also be offered to these individuals.

In conclusion, application of NGS-based gene panel testing for all known FA genes in combination with chromosomal microarrays for *FANCA* or, as a new option—copy number variant (CNV) testing with NGS, provides a comprehensive approach for mutational *FANCA* screening, including identification of large deletions, and should be offered to all clinically diagnosed FA patients. It proves to be a valuable tool for clinical management, including reproductive counseling and prenatal diagnosis for whole families. Identification of novel *FANCA* mutations provides a better understanding of molecular mechanisms and further genotype–phenotype correlation searching of the condition.

## Data Availability

All data generated or analysed during this study are included in this published article.

## Abbreviations

FA: Fanconi anemia
ICL: Interstrand Crosslinking Agents
DEB: Diepoxybutane
MMC: Mitomycin C
aCGH: Array-Comparative Genomic Hybridisation; microarrays
NGS: Next Generation Sequencing
MLPA: Multiplex Ligation-dependent Probe Amplification
PDA: Patent Ductus Arteriosus
CABS: Congenital Abnormality Score
allo-HSCT: Allogenic Hematopoietic Stem Cell Transplant
MUD: Matched Unrelated Donor
GvHD: Graft-versus-Host Disease
CVN: Copy Number Variation
VUS: Variant of Uncertain Significance
